# Supplementary material for: Adherence to European Guidelines for Treatment and Management of Pancreatic Exocrine Insufficiency in Chronic Pancreatitis Patients
Source: J Clin Med. 2021 Jun 21;10(12):2737. doi: 10.3390/jcm10122737 (PMC8233716; doi:10.3390/jcm10122737)
Supplement: Supplementary file 1 [file jcm-10-02737-s001.zip › jcm-1190656-supplementary.pdf]

Table S1: Adherence to each quality indicator in the guideline.

Adherence To Guidelines			
**Quality indicator (QI)**	**Number of patients applicable for guideline**	**Number of patients treated according to guideline**	**Percentage of patients treated according to guidelines (%)**
**Screening for etiology of Chronic pancreatitis**			
1. Alcohol consumption at first visit post 2016	118	77	65.3
2. Alcohol consumption at last visit	114	24	21.1
3. Smoking status at first visit post 2016	118	71	60.1
4. Smoking status at last visit	114	19	16.7
5. Amylase value at PEI diagnosis	118	94	79.7
6. Lipase value at PEI diagnosis	114	82	71.9
7. AIP screening	60	42	70.0
8. Evaluation of family history at time of diagnosis	118	91	77.1
9. Genetic testing if patient is <20 years or has a family history of disease	48	24	50.0
10. Imaging modality used to establish a diagnosis of CP	118	115	97.5
11. MDT-conference prior to first visit or shortly after first visit	118	108	91.5
**Screening for PEI complications**			
Screening for abnormal malnutritional values			
1. Vitamin A at first visit post 2016	118	83	70.3
2. Vitamin A at last visit	114	78	68.4
3. Vitamin E at first visit post 2016	118	81	68.6
4. Vitamin E at last visit	114	76	66.7

5. Vitamin D at first visit post 2016	118	105	89.0
6. Vitamin D at last visit	114	84	73.7
7. Calcium at first visit post 2016	118	79	66.9
8. Calcium at last visit	114	81	71.1
9. Iron at first visit post 2016	118	74	62.7
10. Iron at last visit	114	81	71.1
11. Cobalamine at first visit	118	86	72.9
12. Cobalamine at last visit	114	80	70.1
13. Folate at first visit post 2016	118	59	50.0
14. Folate at last visit	114	76	66.7
15. INR at first visit post 2016	118	95	80.5
16. INR at last visit	114	79	69.3
17. Cholesterol at first visit post 2016	118	103	87.3
18. Cholesterol at last visit	114	73	63.2
19. Thrombocytes at first visit post 2016	118	101	85.6
20. Albumin at first visit post 2016	118	107	90.7
Screening for other PEI complications			
Screening for stool pattern analysis			
1. Stool frequency at first visit post 2016	118	52	44.1
2. Bristol value at first visit post 2016	118	20	16.9
3. Stool frequency at last visit	114	31	27.2
Pain evaluation			
1. Pain evaluation at first visit post 2016	118	99	83.9
Presence of pain		42	
Pain frequency at first visit post 2016	42	18	42.3
Pain intensity at first visit post 2016	42	16	38.1
Pain characteristics at first visit post 2016	42	16	38.1
2. Pain evaluation at last visit	114	73	64.0
Presence of pain		29	
Pain frequency at last visit	29	13	44.8
Pain intensity at last visit	29	8	27.6
Pain characteristics at last visit	29	9	31.0
Screening for osteoporosis			

1. Dexa performed at diagnosis and least once in the last 24 months if diagnosed with osteoporosis	118	55	46.6
Screening for BMI			
1. BMI at first visit post 2016	118	81	68.6
2. BMI at last visit	114	42	36.8
Screening for endocrine insufficiency			
1. HbA1c at first visit post 2016	118	80	67.8
2. HbA1c at last visit	114	75	65.7
**Therapy of CP complications**			
1. PERT given according to guidelines	118	101	85.6
Therapy of malnutritional values			
1. Increase of PERT dosage if patient shows symptoms	29	18	62.1
2. Addition of PPI	18	6	33.3
3. Vitamin A	8	2	25.0
4. Vitamin E	10	4	40.0
5. Vitamin D	11	8	72.7
6. Calcium	5	3	60.0
7. Iron	16	5	31.3
8. Cobalamine	18	14	77.8
9. Folate	6	5	83.3
Treatment of other PEI complications			
1. Pain therapy according to WHO pain ladder	42	28	66.7
2. Treatment of osteoporosis	20	16	80.0
3. Visit to dietitian	118	18	15.2
Treatment of diabetes mellitus			
1. Application of DM therapy	34	29	85.3
